# G6PD deficiency, primaquine treatment, and risk of haemolysis in malaria-infected patients

**DOI:** 10.1186/s12936-018-2564-2

**Published:** 2018-11-08

**Authors:** Sara Avalos, Rosa E. Mejia, Engels Banegas, Cesar Salinas, Lester Gutierrez, Marcela Fajardo, Suzeth Galo, Alejandra Pinto, Angel Mejia, Gustavo Fontecha

**Affiliations:** 1 Microbiology Research Institute, National Autonomous University of Honduras, Tegucigalpa, Honduras; 2 Pan American Health Organization, Tegucigalpa, Honduras; 3 National Department of Surveillance, Ministry of Health, Tegucigalpa, Honduras

**Keywords:** G6PD, Primaquine, Haemolysis, Honduras

## Abstract

**Background:**

The incidence of malaria in the Americas has decreased markedly in recent years. Honduras and the other countries of Mesoamerica and the island of Hispaniola have set the goal of eliminating native malaria by the year 2020. To achieve this goal, Honduras has recently approved national regulations to expand the possibilities of a shortened double dose primaquine (PQ) treatment for vivax malaria. Considering this new shortened anti-malarial treatment, the high frequency of G6PDd genotypes in Honduras, and the lack of routinely assessment of the G6PD deficiency status, this study aimed at investigating the potential association between the intake of PQ and haemolysis in malaria-infected G6PDd subjects.

**Methods:**

This was a prospective cohort and open-label study. Participants with malaria were recruited. *Plasmodium vivax* infection was treated with 0.25 mg/kg of PQ daily for 14 days. Safety and signs of haemolysis were evaluated by clinical criteria and laboratory values before and during the 3rd and 7th day of PQ treatment. G6PD status was assessed by a rapid test (CareStart™) and two molecular approaches.

**Results:**

Overall 55 participants were enrolled. The frequency of G6PD deficient genotypes was 7/55 (12.7%), where 5/7 (71.4%) were hemizygous A− males and 2/7 (28.6%) heterozygous A− females. Haemoglobin concentrations were compared between G6PD wild type (B) and G6PDd A− subjects, showing a significant difference between the means of both groups in the 3rd and 7th days. Furthermore, a statistically significant difference was evident in the change in haemoglobin concentration between the 3rd day and the 1st day for both genotypes, but there was no statistical difference for the change in haemoglobin concentration between the 7th day and the 1st day. Besides these changes in the haemoglobin concentrations, none of the patients showed signs or symptoms associated with severe haemolysis, and none needed to be admitted to a hospital for further medical attention.

**Conclusions:**

The findings support that the intake of PQ during 14 days of treatment against vivax malaria is safe in patients with a class III variant of G6PDd. In view of the new national regulations in the shortened treatment of vivax malaria for 7 days, it is advisable to be alert of potential cases of severe haemolysis that could occur among G6PD deficient hemizygous males with a class II mutation such as the Santamaria variant, previously reported in the country.

**Electronic supplementary material:**

The online version of this article (10.1186/s12936-018-2564-2) contains supplementary material, which is available to authorized users.

## Background

Malaria remains one of the diseases with higher rates of morbidity and mortality in the tropical world. According to the World Health Organization (WHO) an estimated of 216 million cases of malaria occurred worldwide in 2016, however the incidence rate of malaria is estimated to have decreased by 18% globally between 2010 and 2016, with the WHO Region of the Americas recording the second largest decline (22%) after the South-East Asia Region (48%) [1]. The incidence of malaria cases has decreased in Honduras by 96% since 2000, and together with the countries of Mesoamerica and the Hispaniola Island, the goal of eliminating native malaria by the year 2020 has been proposed [2]; however, a more realistic projection includes Honduras among the countries that could reduce the incidence of cases by ≥ 40% by 2020 hereinafter [1].

The only two parasite species causing malaria in Honduras are *Plasmodium vivax* and *Plasmodium falciparum*, responsible for 90% and 10% of malaria cases, respectively (pers. comm. National Surveillance Laboratory of the Ministry of Health). Due to persistent liver stages (hypnozoites) of *P. vivax* relapses of the disease may occur weeks or months after the first infection [3, 4]. It is estimated that most of the malarial attacks caused by *P. vivax* have their origin in the reactivation of hypnozoites [5], that can only be prevented by 8-aminoquinoline anti-malarials, such as primaquine (PQ) [6]. However, the greatest limitation in the use of this drug is the risk of haemolysis in patients with a significant enzymatic deficiency of G6PD [7].

G6PD deficiency is an X-linked genetic disorder that affects more than 400 million people worldwide, and its average prevalence in countries with endemic malaria is 8% (3–30%) [8, 9]. Recent data indicate a high frequency of the class III African variant A− (11.81%) among the Honduran population living in malaria endemic municipalities [10]. PAHO states that the intake of PQ for the treatment of vivax malaria is safe in all cases when dosed for 14 days, even in individuals with the African A− variant [9, 11]. In contrast, subjects with more severe G6PDd variants (e.g. Mediterranean, Santamaria), could suffer of haemolysis as a life-threatening condition if PQ intake is not stopped or if it is administered at high doses [12, 13]. In Honduras, the current national regulations for malaria treatment indicates the ambulatory use of PQ in doses of 0.25 mg/kg daily for 14 days for vivax infections, or 0.75 mg/kg single dose for falciparum infections, but no previous diagnosis of G6PDd is performed in any case [14].

Because of the new goals to eliminate malaria from the Mesoamerican/Hispaniola sub-region by 2020, the new national regulation of Honduras approved in 2017 opens the possibility of administering PQ in a shortened double dose scheme for 7 days for vivax malaria to improve the adherence to treatment. Although shortening treatment could improve patients’ adherence in order to a radical cure of the parasite, it could also increase the risk of haemolysis in individuals with G6PDd, particularly those hemizygous males affected with severe class II variants.

Therefore, when considering the high frequency of G6PDd genotypes in Honduras, the blind administration of PQ without G6PDd analysis at the point of care, and the change in the PQ treatment scheme from 14 to 7 days at double dose, it is pertinent to investigate if there is an association between the intake of primaquine and haemolysis in G6PD deficient subjects infected with malaria.

## Methods

### Settings, study design and participants

**Fig. 1:**
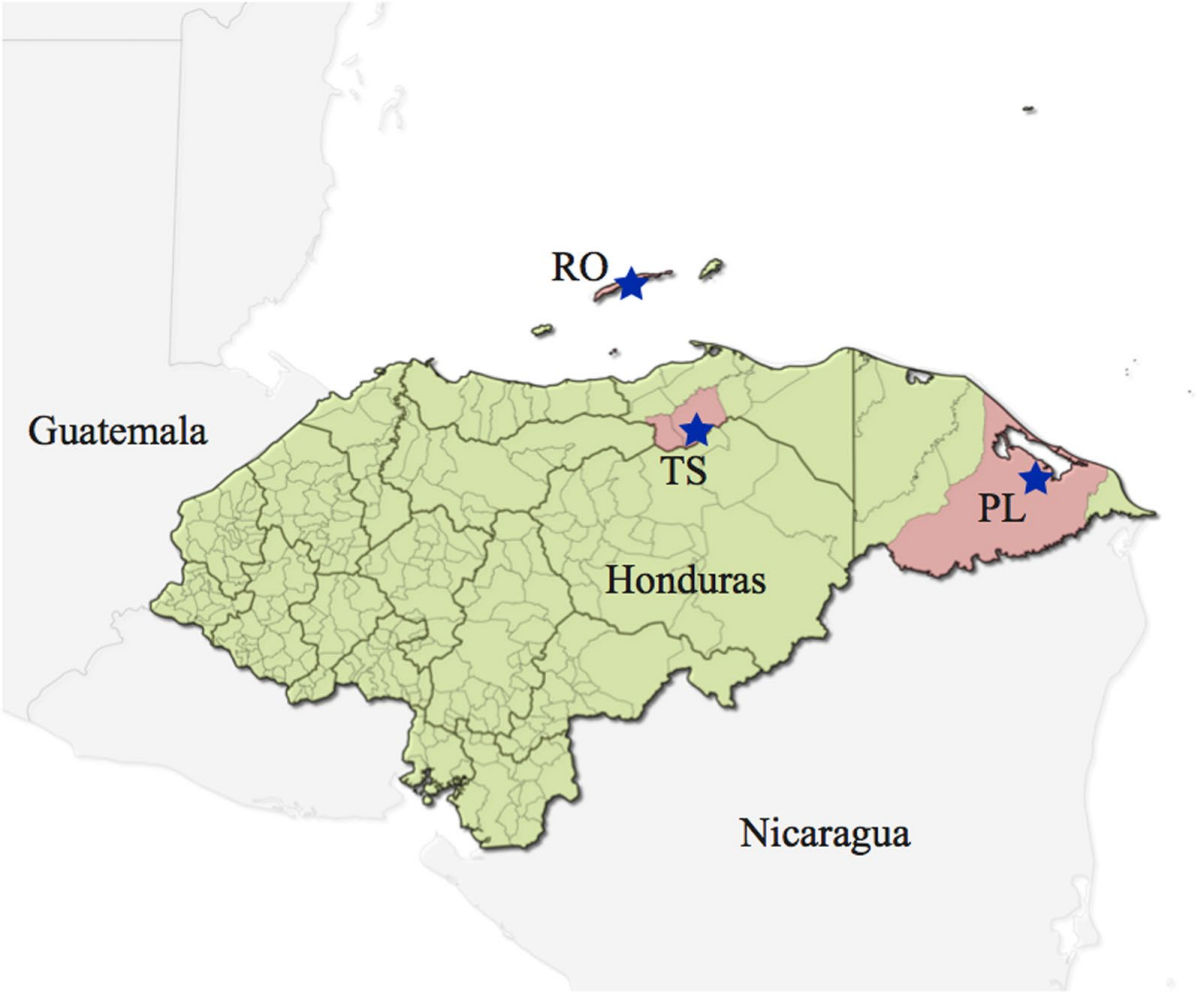
Map of Honduras locating the three selected cities where samples were collected. Roatan (RO), Tocoa and Saba (TS), and Puerto Lempira (PL)

This was a prospective cohort and open-label study carried out between February and June 2017. Participants were recruited from 4 malaria endemic municipalities located at the northern coast of Honduras: Tocoa/Saba (Department of Colon), Roatan (Department of Bay Islands), and Puerto Lempira (Department of Gracias a Dios) (Fig. 1). Male and female individuals over 10 years old were included as long as they presented current malarial infection and had not started treatment. Participants requested health care in national centres or were recruited through active case detection. Pregnant women, breastfeeding or patients under previous anti-malarial treatment were not included.

### Trial procedures

**Fig. 2:**
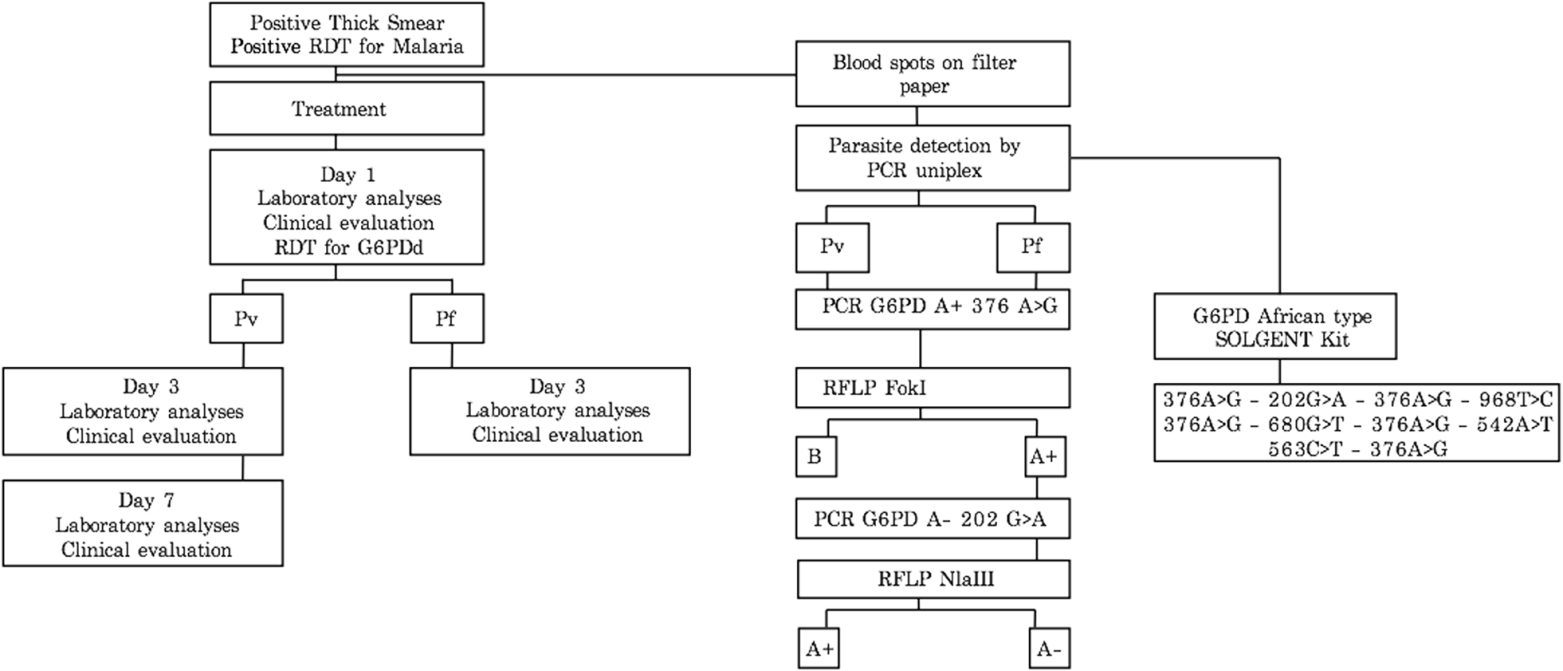
Flow diagram illustrating the methods, procedures and follow-ups carried out in this study

The microscopic diagnosis of parasitaemia was performed by thick blood according to national standard procedures and confirmed by the immunochromatographic test RAPID 1-2-3^®^ HEMA Cassette Malaria PF/PV (Miramar, FL, USA). A flowchart showing the procedures carried out in this study can be found in Fig. 2.

Immediately after the malaria diagnosis a phenotypic test was performed for the detection of G6PD deficiency (CareStart™, ACCESS BIO INC., NJ, USA). Patients were treated according to the National Malaria Standard valid since 2010 [14]. Uncomplicated vivax infections received 25 mg/kg of chloroquine (CQ) spread over 3 days plus 0.25 mg/kg of primaquine (PQ) daily for 14 days. Patients infected with *P. falciparum* received 25 mg/kg of CQ over 3 days plus 0.75 mg/kg of PQ in a single dose the first day of treatment.

Patients were clinically assessed the first day of medical consultation, and during treatment. Two follow-ups were conducted to each participant by trained physicians, visiting them in their households. Blood samples were collected on the 1st day before treatment, and during the 3rd and 7th day of PQ treatment. Patients infected with *P. falciparum* were visited and monitored only the 3rd day of treatment. The clinical evaluation determined vital signs such as heart rate, respiratory rate, temperature and blood pressure. Symptoms and signs such as low back pain, abdominal pain, choluria, oliguria, jaundice, respiratory distress, pallor, and hepatosplenomegaly, were also recorded. Blood samples were sent to local laboratories to quantify levels of haemoglobin, haematocrit, red blood cells, reticulocytes, AST, ALT, bilirubin and creatinine. The values of reticulocytes, AST, ALT, bilirubin and creatinine could not be obtained for the participants of Puerto Lempira due to the lack of an appropriate clinical laboratory in the city.

### DNA extraction and molecular methods

A drop of blood was taken on filter paper from each participant on the first day of recruitment for further molecular analyses. Genomic DNA was extracted using a Chelex-100 based method [15]. To confirm the microscopic diagnosis of malaria and the parasite species, single-tube species-specific PCRs were carried out using primers AL7178/AL7142 and AL7175/AL7074 according to previously described reports [16].

In order to identify G6PD genotypes associated with enzyme deficiency, two molecular approaches were carried out, an in-house PCR–RFLP and the DiaplexC™ G6PD genotyping (African type) commercial kit (Solgent Co., Ltd. Seoul, Korea). The PCR–RFLP assay was designed to independently identify single nucleotide polymorphisms in the positions 202 (G→A) and 376 (A→G) within the gene corresponding to exons IV and V. This method was carried out according to protocols previously described and adapted [10, 17, 18]. This restriction analysis allowed to detect two of the most common African G6PD variants, G6PD A+ considered as class IV with normal enzyme activity, and the class III variant G6PD A− with 10–60% residual enzyme activity [19]. The Solgent^®^ commercial kit enabled the detection of the following six variants: Class IV variant A+ (376A→G); 3 different class III variants A− (376A→G; 202G→A), (376A→G, 968T→C), (376A→G; 680G→T); class II Santamaria (376A→G, 542A→T); and class II Mediterranean (376A→G, 563C→T). This procedure was carried out according to manufacturer’s instructions.

Additionally, a genetic analysis of the β-globin gene was also performed to reveal the presence of the sickle cell trait (β^S^) among the studied population. A PCR–RFLP approach was conducted according to previous reports [20, 21].

### Statistical analysis

Data were entered and verified using Microsoft Excel™ software, and analysed using IBM SPSS Statistics for Windows, version 22.0 (Armonk, NY: IBM Corp.). The Kolmogorov–Smirnov (n ≥ 50) and Shapiro–Wilk (n < 50) tests were used to evaluate whether the data were normally distributed for each laboratory variable (haemoglobin, haematocrit, red blood cells, reticulocytes, AST, ALT, bilirubin and creatinine) within each group (genotype). A *p* value ≥ 0.05 revealed normal distribution within each genetic group for the evaluated variables. The variables that did not meet normality criteria could not be analysed by Student’s t test. Student’s t test was used to compare the mean values of normally distributed data among individuals with deficient G6PD genotype and those with normal genotype.

The change in concentration for normally distributed data was expressed in absolute values. The difference among values recorded on the 3rd and 7th day of primaquine intake with respect to the baseline value of the 1st day before treatment was calculated for each group. The distributions of these changes were graphically represented by box plots. In all the statistical tests a *p* value < 0.05 was established for the rejection of the null hypothesis.

The measure of agreement between G6PD molecular techniques (PCR–RFLP and Solgent^®^ Kit) was assessed by the Cohen’s kappa coefficient using the Epidat program, v. 3.1. A *p* value = 0.05 and confidence intervals = 95% were considered. The agreement between the RDT and the molecular approaches could not be calculated since the RDT is able to detect G6PD deficiencies with less than 10% of enzyme activity, and the most common class III mutations detected through the PCR–RFLP and the multiplex PCR are associated with residual enzyme activity of 10–60%.

## Results

### Study population and malaria infections

From February to May 2017, fifty-five patients with acute malaria were enrolled from 4 endemic municipalities of Honduras (Tocoa/Saba, n = 5; Puerto Lempira, n = 10; Roatan, n = 40). Thirty-three patients were males and twenty-two females. Ten participants were under 18 years old, seven of them from Puerto Lempira. Ninety-six percent of infections were caused by *P. vivax*, and the remaining 3.6% by *P. falciparum* (Table 1). The microscopic diagnosis was confirmed by a rapid diagnostic test (RDT) and a molecular approach, with 100% of agreement.

**Table 1:** Number of malaria infections according to parasite species, locality, and sex

Locality	N	Sex	Total (%)	*P. vivax* (%)	*P. falciparum* (%)
Tocoa/Saba	5	M	3 (5.4)	2 (3.6)	1 (1.8)
		F	2 (3.6)	2 (3.6)	
Puerto Lempira	10	M	5 (9.1)	4 (7.3)	1 (1.8)
		F	5 (9.1)	5 (9.1)	
Roatan	40	M	25 (45.5)	24 (43.6)	
		F	15 (27.3)	16 (29.1)	
Total	55	M	33 (60.0)	30 (54.5)	2 (3.6)
		F	22 (40.0)	23 (41.8)	

### Prevalence of G6PD deficiency

The patients were screened for G6PD deficiency through a phenotypic and qualitative point-of-care test at the time of enrolment. This RDT relies on a visual interpretation of colour change that classifies individuals as normal or deficient. According to the manufacturer this method is well-suited for deficient patients with less than 10% of enzymatic activity (variants class I and II). The phenotypic test detected 5 (9.1%) deficient patients, 4 of them males (Table 2). All patients were also tested by two genotypic methods for the detection of G6PD mutations, an in-house PCR–RFLP and a commercial multiplex PCR. The PCR–RFLP assay was intended for detecting 2 SNPs associated with A+ and A− deficient genotypes. Seven subjects (12.7%) were found to be A− and none A+. Five of seven patients with A− genotype were hemizygous males and two heterozygous females. The age range of the five male participants with genotype A−  was 12–61 years (average = 29.6; SD = 22.5). Subjects with the A− deficient genotype were found in Tocoa, Puerto Lempira and Roatan. The DiaplexC™ G6PD commercial kit (Solgent^®^) is able to detect six mutations (1 class IV, 3 class III, and 2 class II variants) and revealed six subjects with genotype A− (all mutations were: 376A→G; 202G→A) and one with genotype A+ (376A→G) (Table 3).

**Table 2:** Phenotypic analysis of G6PD deficiency by CareStart™, and comparison of genotyping results between PCR–RFLP and the DiaplexC™ G6PD commercial kit (Solgent^®^)

	RDT		PCR–RFLP			Solgent Kit		
	Normal	Deficient	B	A+	A− ^a^	B	A+	A−
Male	29	4	28	–	5	28	1	4
Female	21	1	22	–	–	20	–	2
Total	50	5	50	–	5	48	1	6

^a^Hemizygous males

**Table 3:** Genotyping of G6PD deficiency by location and sex, performed by a PCR–RFLP method

Locality	N	G6PD genotype	N (%)	Heterozygous female	Homozygous female	Hemizygous male
Tocoa/Saba	5	A− ^a^	1 (1.8)	–	–	1 (1.8)
		A+ ^b^	–	–	–	–
		B^c^	4 (7.3)	–	–	–
Puerto Lempira	10	A−	3 (5.5)	1 (1.8)	–	2 (3.6)
		A+	–	–	–	–
		B	7 (12.7)	–	–	–
Roatan	40	A−	3 (5.5)	1 (1.8)	–	2 (3.6)
		A+	–	–	–	–
		B	37 (67.3)	–	–	–
Total	55	A−	7 (12.7)	2 (3.6)	–	5 (9.1)
		A+	–	–	–	–
		B	48 (87.3)	–	–	–

^a^A− (376A→G; 202G→A)

^b^A+ (376A→G)

^c^Wild type

The results of the two genetic tests matched on the diagnosis of the A− genotype for 2 heterozygous women and 4 of 5 hemizygous males. However, one of the subjects with genotype A− by PCR–RFLP revealed a genotype A+ with the commercial kit revealing a Kappa index = 0.723 (IC 95% = 0.451, 0.995), p < 0.001. When comparing the results of the genotypic and phenotypic approaches, we found that only 2 phenotypically deficient patients showed a class III A− variant (hemizygous males). In contrast, 3 patients with phenotypic G6PD deficiency by RDT showed a normal B genotype; and 5 patients (4 males and 1 heterozygous female) with a normal phenotype were classified as A− by PCR–RFLP. All discrepant genetic tests were repeated with the same results. The discordant phenotypic tests could not be repeated because the genetic results were obtained months later, and fresh blood samples were required.

### Clinical assessment of haemolysis symptoms

All the participants were clinically evaluated the first day of enrolment and the 3rd day of primaquine (PQ) treatment. Patients with vivax malaria (n = 53) were also evaluated on the 7th day of treatment. Signs and symptoms suggestive of haemolysis were recorded as present or absent. None of the patients showed signs or symptoms associated with severe haemolysis, and none needed to be admitted to a hospital for further medical attention. Mild or moderate abdominal pain was the most commonly reported sign, followed by hepatomegaly (Table 4). There was no evidence of an increase in the severity of the symptoms throughout the treatment, and neither there was no notable difference between the deficient and non-deficient patients regarding the number of individuals reporting adverse effects.

**Table 4:** Number of subjects with G6PD A and B genotypes adversely affected after primaquine treatment

Symptoms	G6PD A− males (n = 5)			G6PD A (n = 7)			G6PD B (n = 48)		
	Day 1 n (%)^a^	Day 3 n (%)	Day 7 n (%)	Day 1 n (%)^a^	Day 3 n (%)	Day 7 n (%)	Day 1 n (%)^a^	Day 3 n (%)	Day 7 n (%)
Jaundice	2 (40.0)	1 (20.0)	–	2 (28.6)	2 (28.6)	–	5 (10.4)	3 (6.3)	–
Respiratory difficulty	–	–	–	–	–	–	3 (6.3)	2 (4.2)	2 (4.2)
Bleeding	–	–	–	–	–	–	1 (2.1)	1 (2.1)	1 (2.1)
Edema	–	–	–	–	–	–	–	–	–
Abdominal pain	2 (40.0)	–	–	2 (28.6)	2 (28.6)	1 (14.2)	12 (25.0)	13 (27.1)	1 (2.1)
Hepatomegaly	2 (40.0)	–	–	3 (42.9)	3 (42.9)	2 (28.5)	10 (20.8)	5 (10.4)	1 (2.1)
Splenomegaly	2 (40.0)	–	–	2 (28.6)	1 (14.3)	1 (14.2)	2 (4.2)	1 (2.1)	–
Low back pain	–	–	–	–	–	1 (14.2)	17 (35.4)	8 (16.7)	3 (6.3)
Pallor	2 (40.0)	1 (20.0)	1 (20.0)	2 (28.6)	2 (28.6)	1 (14.2)	–	–	–

^a^Before primaquine treatment

### Haematological and biochemical changes after primaquine treatment

In order to evaluate haemolysis levels during treatment with PQ, haematological analyses were performed to all participants (n = 55) during the 1st, 3rd and 7th follow-up days; however, biochemical analyses were performed only to participants who resided in Tocoa/Saba and Roatan (n = 45) due to logistic limitations in Puerto Lempira city.

Table 5 shows the means of the laboratory parameters and compares these values in the group of patients with wild genotype (B) with those of deficient genotypes (A− males and A).

**Table 5:** Mean of haematological and biochemical parameters of G6PD (genotypes A) deficient and non-deficient (genotype B) subjects taking primaquine for 3 and 7 days

	Day 1: mean (SD) (n)^a^			Day 3: mean (SD) (n)			Day 7: mean (SD) (n)		
	A− (males)	A	B	A− (males)	A	B	A− (males)	A	B
RBC × 10^6^/µL	4.03 (0.42) (5)	4.1 (0.7) (7)	4.4 (0.5) (48)	3.7 (0.5) (5)	3.6 (0.7) (7)	4.1 (0.6) (48)	3.7 (0.3) (5)	3.9 (0.5) (6)	4.2 (0.5) (46)
Haemoglobin g/dL	11.3 (1.71) (5)	11.6 (2.0) (7)	12.4 (1.7) (48)	10.0 (1.3) (5)	10.2 (1.9) (7)	11.8 (1.7) (48)	9.9 (1.6) (5)	10.7 (1.9) (6)	12.1 (1.4) (46)
Hematocrit %	34.5 (5.05) (5)	35.4 (6.2) (7)	37.5 (5.0) (48)	30.4 (3.2) (5)	30.9 (5.4) (7)	36 (4.7) (48)	29.4 (4.7) (5)	32.1 (6.6) (6)	36.8 (4) (46)
Reticulocyte %	1.1 (0.5) (3)	1.1 (0.5) (4)	2. 2 (1.8) (41)	2.4 (1.3) (3)	2.2 (1.2) (4)	2.6 (1.9) (41)	11.2 (9.5) (3)	11.2 (9.5) (3)	3.6 (2.9) (40)
AST IU/L	30.1 (24.2) (3)	29.2 (19.8) (4)	34.6 (19.7) (41)	35.3 (7.2) (3)	32.6 (7.9) (4)	31.8 (21.5) (41)	39.0 (33.9) (3)	39.0 (33.9) (3)	36.9 (25.4) (40)
ALT IU/L	33.6 (33.1) (3)	30.3 (27.8) (4)	37.2 (24.9) (41)	29.4 (17) (3)	27.1 (14.6) (4)	37.2 (31) (41)	120.2 (113) (3)	120.2 (113) (3)	52.3 (48.3) (40)
Total bilirubin mg/dL	1.2 (0.6) (3)	1.1 (0.6) (4)	1.4 (0.8) (41)	2.0 (1.1) (3)	1.8 (1.0) (4)	0.9 (0.4) (41)	0.6 (0.2) (3)	0.6 (0.2) (3)	0.7 (0.31) (40)
Direct bilirubin mg/dL	0.8 (0.6) (3)	0.7 (0.5) (4)	0.9 (0.7) (41)	1.1 (0.7) (3)	0.9 (0.7) (4)	0.6 (0.3) (41)	0.3 (0.1) (3)	0.3 (0.1) (3)	0.5 (0.2) (40)
Indirect bilirubin mg/dL	0.4 (0.2) (3)	0.4 (0.2) (4)	0.6 (0.4) (41)	0.9 (0.7) (3)	0.9 (0.6) (4)	0.4 (0.3) (41)	0.3 (0.1) (3)	0.3 (0.1) (3)	0.3 (0.2) (40)
Creatinin mg/dL	1.0 (0.2) (3)	1.0 (0.13) (4)	0.9 (0.1) (41)	0.9 (0) (3)	0.9 (0) (4)	0.8 (0.1) (41)	0.9 (0.1) (3)	0.9 (0.1) (3)	0.8 (0.2) (40)

^a^Before primaquine treatment

The mean of red blood cells counts, haemoglobin, and haematocrit percentage were lower than 4.4 × 10^6^/μL, 12.4 g/dL, and 37.5%, respectively, during the follow-up of days 3 and 7 and for both genetic groups. The mean of reticulocyte percentage was higher than 1% in all cases. The results of the biochemical analyses were more similar to normal values, with the exception of a slight elevation in bilirubin.

Normality in the distribution of laboratory results was evaluated within each genotype (A− and non-A−). Only haemoglobin values revealed a normal distribution of data (Kolmogorov–Smirnov test: *p* ≥ 0.05) within each group and could be analysed by Student’s t test. The rest of the laboratory data were not analysed by the Student’s t test. The mean haemoglobin concentrations on the 1st day did not show a statistically significant difference between the A− and non-A− genotypes, however, there was a significant difference between the means of both groups in the 3rd and 7th days, with *p* values < 0.05 (Table 6).

**Table 6:** Mean and *p* value of haemoglobin concentration by G6PD genotype during 7 days of primaquine intake

Day of PQ treatment	G6PD genotype	n	Mean of haemoglobin concentration g/dL (SD)	p*
1^a^	A−	5	11.30 (1.71)	0.191
	Non-A−	50	12.39 (1.75)	
3	A−	5	9.98 (1.29)	0.034
	Non-A−	50	11.74 (1.76)	
7	A−	4^b^	9.88 (1.63)	0.004
	Non-A−	48^b^	12.07 (1.40)	

*p values of Student’s t comparing means of two independent samples

^a^Before primaquine treatment

^b^Missing data of participants with G6PD A genotype on day 7 of treatment (*P. falciparum* infection)

**Fig. 3:**
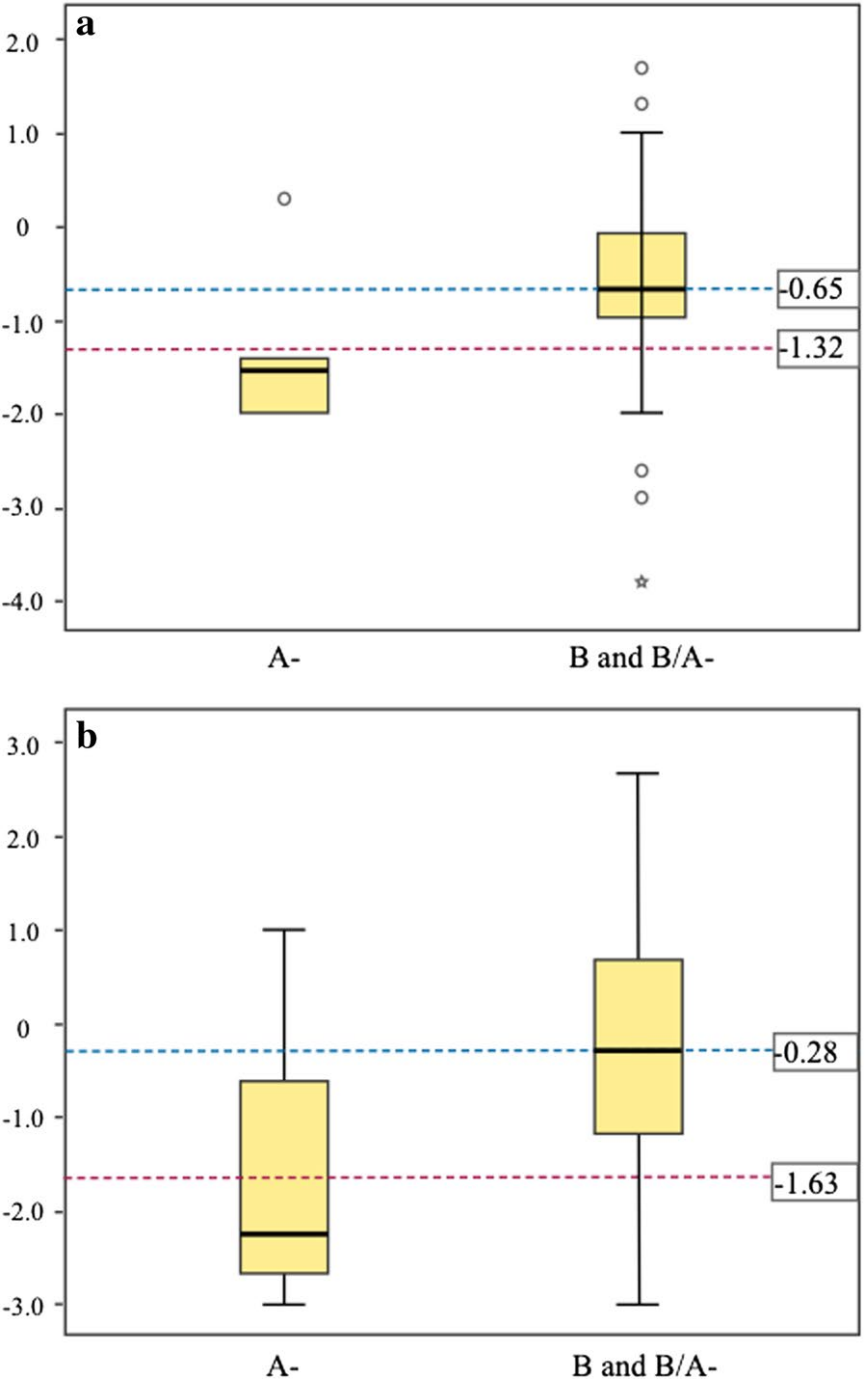
Box plots showing the distribution of absolute changes in haemoglobin concentration of G6PD deficient and non-deficient subjects on the 3rd day (**a**), and the 7th day (**b**), relative to basal levels before primaquine treatment on day 1. The dotted line indicates median. The Y axis indicates the absolute loss of haemoglobin (g/dL). Genotypes A (deficient) and B (normal) are indicated in the X axis

The difference was also calculated in the haemoglobin concentration among the values recorded during the 3rd day and 7th day with respect to the 1st day before PQ treatment for each genotype and compared the means of those changes by Student´s t test. As shown in Table 7, a statistically significant difference was shown in the change in haemoglobin concentration between the 7th day and the 1st day for both genotypes (*p *= 0.040). The distributions of these changes are shown in Fig. 3. Supplementary information regarding the haemoglobin concentration of individuals by age and sex with a non-deficient G6PD genotype is showed in Additional file 1: Table S1.

**Table 7:** Absolute changes in haemoglobin levels on days 3 and 7 during follow-up relative to the enrolment (1st day) for G6PD deficient and non-deficient subjects

Comparison between days	G6PD genotype	*n*	Mean (SD)	p*
Day 3–Day 1	A−	5	− 1.32 (0.95)	0.157
	Non-A−	50	− 0.65 (1.00)	
Day 7–Day 1	A−	4^a^	− 1.63 (1.79)	0.040
	Non-A−	48^a^	− 0.28 (1.18)	

*p values of Student’s t comparing means of two independent samples

^a^Missing data of participants on day 7 of treatment (*P. falciparum* infection)

The results of the genetic analysis of sickle cell trait revealed two heterozygous (β^S^/β^A^) women (3.6%), one of them heterozygous for the G6PD A− mutation and the other one with a G6PD wild genotype.

## Discussion

In order to evaluate the risk of haemolysis in patients infected with malaria and treated with primaquine in Honduras, fifty-five subjects were enrolled and treated according to the national scheme in force. The patients were recruited from three departments with the highest endemicity in the country, accounting for almost 70% of cases (Colon = 18.6%; Gracias a Dios = 33.4%; Bay Islands = 17.6%). The number of infections caused by *P. vivax* was 53 (96.4%) and the remaining 3.6% by *P. falciparum*. These results are consistent with the official data for the year 2017 in Honduras, which indicate 1155 cases (90%) for vivax malaria and the remaining 10% caused by *P. falciparum*.

According to the CareStart™ rapid diagnostic test (RDT), 5 (9.1%) subjects were classified as deficient, however only 2 of these 5 subjects revealed a hemizygous A− genotype through molecular analyses of the G6PD gene. In contrast, the other three subjects with putative phenotypic deficiency showed a wild genotype B.

Although several authors report acceptable percentages of sensitivity, specificity, and predictive values for the detection of G6PD deficiency by the CareStart™ test [22–25], there is also a minority of reports showing some kind of low performance for this RDT [26, 27]. In Cambodia the performance of the CareStart™ RDT was compared to a quantitative spectrophotometric method, and 1.4% of subjects were falsely classified as normal [27]. In Manaus, male participants with less than 30% of enzyme activity were assessed through CareStart™ RDT with only 61.5% sensitivity [26]. The possible reasons behind these apparent discrepancies in some results of the CareStart™ test have been clearly addressed by Monteiro et al. [28]. In the present study, as suggested by those authors, the performer´s subjectivity in the interpretation of the RDT results could be the main cause for the discrepancy between techniques, since the difference between a purple colour, faint purple, and no colour could lead to confusion for unskilled eyes. Another less plausible explanation is the presence of undetected G6PD mutations in those subjects classified as deficient by RDT. On the other hand, 5 subjects with a normal result by RDT were classified as A− by PCR–RFLP. This could be due to a detection threshold of < 10% in the enzyme activity for the RDT (with > 95% sensitivity for G6PDd variants I and II) [29], while individuals with a class III variation (i.e. genotype A−) normally have G6PD activity higher than 10%. Given the discrepancy between the RDT and the genetic analyses, and because the most frequent (16.08%) deficient genotypes in Honduras are classified as “A” (A− class III, and A+ class IV variants) [10], for further analyses the participants in this study were classified as “deficient” when they had the “A−” genotype, or “normal” when a wild genotype B or A+ (non-A−) genotype were confirmed according to the PCR–RFLP results.

In order to detect clinical signs associated with haemolysis, the 55 participants were evaluated before the start of anti-malarial treatment and on the 3rd day of treatment. Most of them (n = 53) were also evaluated on the 7th day of treatment. None of the patients showed signs associated with severe haemolysis and there was no notable difference between patients with wild genotype and those with deficient genotype. As established by the WHO [30], this result seems to confirm the safety of the intake of 0.25 mg/kg of PQ daily for 14 days in vivax infections, or 0.75 mg/kg of PQ single dose in falciparum infections, for individuals without G6PD deficiency and for those with class III and IV deficiency.

Similar results were observed when analysing haematological values from patients during PQ treatment. As shown in Table 5, mean levels of haemoglobin, haematocrit, RBC and reticulocytes before the start of PQ treatment are indicative of mild anaemia [31] in both groups (deficient and normal), with slightly lower mean levels in deficient patients. This mild anaemia could be attributed to both the intrinsic pathogenesis of the parasitic infection [32] as to the high baseline prevalence of anaemia in the Honduran population, estimated at 17.8% among women of reproductive age, and 31.4% among children under 5 years [33].

A previous report indicates that more than 23% of the Afro-descendant population living in malaria endemic areas in Honduras presents the sickle-cell trait, and an overall prevalence of anaemia of 47% in 2014 [21]. Even though no association between the sickle cell trait and anaemia was observed then [21], patients were analysed in search of β^S^ alleles. These findings seem to rule out the influence of sickle cell disease as a contributor to mild anaemia in this study, since only 2 patients were heterozygous for β^S^.

The results of the biochemical tests did not reveal a clear elevation of bilirubin or hepatic enzymes derived from treatment, which correlates well with the lack of jaundice or hepatomegaly in most patients. In order to establish a statistically significant difference between the means of the laboratory values of patients with normal genotype and those with deficient genotype, normality in the distribution of data was assessed. Possibly due to the small number of the sample, only the haemoglobin values revealed normal distribution and could be analysed by Student’s t test. A significant difference between the means of haemoglobin values for the normal and the deficient subjects was established after the start of PQ treatment (*p* = 0.034 for day 3, and *p* = 0.004 for day 7) (Table 6). A statistically significant difference was also shown in the change in haemoglobin concentration between the 7th day and the 1st day was found (Table 7).

These results could mean that patients with deficient genotype suffer a greater loss of haemoglobin than patients without G6PD deficiency due to the intake of PQ. According to the WHO [31], anaemia is classified as moderate in people older than 15 years when haemoglobin reaches values between 8.0 and 10.9 g/dL. According to that scale of anaemia, the mean of haemoglobin for deficient patients was 10.0 and 9.9 g/dL during days 3 and 7 of PQ treatment, respectively, which would be considered as moderate anaemia. In contrast, the mean haemoglobin for non-deficient subjects would be classified as mild anaemia or without anaemia.

Most studies evaluating the safety of PQ intake in G6PDd patients are based on *P. falciparum* infections receiving a single dose of PQ administered at different concentrations (0.25 or 0.4 mg/kg [34, 35]; 0.4–0.5 mg/kg [36]; 0.1, 0.4, 0.75 mg/kg [37]; 0.7 mg/kg [38]). Those studies seem to agree on two points: that the adverse effects of PQ at low doses are usually mild or moderate even in patients with G6PDd; and that safety depends primarily on primaquine doses, as well as the variant of G6PD deficiency.

Studies evaluating the safety of PQ in G6PDd patients infected by *P. vivax* requiring longer treatments (7–14 days) are scarcer. A study conducted in Thailand compared the PQ safety (30 mg once daily for 7 days) in G6PD deficient and not deficient patients. A significant reduction in haematocrit was recorded only in patients with G6PDd after the 7th day of treatment, but without triggering severe anaemia [39]. Unfortunately, this study classified the patients as deficient through a screening test without evaluating the genetic variants of G6PD. A second study assessed quantitatively the haemolytic risk of tafenoquine (TQ) or PQ (15 mg for 14 days) in normal and heterozygous women carrying the G6PDd Mahidol (Class III variant) and an enzyme activity between 40 and 60%. In the primaquine arm of the study, haemoglobin levels did not fall below 9 g/dL in any subject, but 3 out of 5 participants showed haemolytic events following PQ treatment (haemoglobin decline of > 2.5 g/dL) [40]. With similar results, Chu et al. [41] evaluated two PQ treatments (0.5 mg/kg daily for 14 days versus 1.0 mg/kg daily for 7 days) in G6PDd heterozygous women with the Mahidol variant and vivax malaria. In that study 0.5 mg/kg of PQ were reasonably well tolerated but there was an association between PQ doses and haemolysis, with mean haematocrit reductions of 20.4% when PQ was administered at the higher dose (1.0 mg/kg for 7 days). Those authors suggest that monitoring the treatment is indispensable when daily doses of PQ greater than 0.5 mg/kg are administered.

In the Honduran context, the malaria caused by *P. vivax* constitutes 90% of annual cases, and its treatment requires 14 days of treatment with PQ in doses of 0.25 mg/kg [14]. In addition, the most common variants of G6PDd in Honduras are class III and IV, and thus it would not seem necessary to evaluate the G6PD status of the patients before administration of PQ. However, there are some reports of severe anaemia, hospitalization, and even death in G6PDd patients treated with low doses of PQ [35, 38, 41–43]. Most of cases are due to more severe variants of G6PDd. In Honduras, there is a previous report of the Santamaria variant classified as class II [10]. Circumstantially, during the enrolment of participants for this study, a second patient with the G6PDd Santamaria variant was detected in the country. A 16-year-old male patient with vivax malaria, who did not participate in this study, was treated daily with 30 mg of PQ. Seventy-two hours after starting the treatment, vomiting, respiratory distress, melaena, haemoglobinuria, painful hepatosplenomegaly, severe anaemia, renal failure and severe generalized jaundice occurred. Misdiagnosis of severe malaria was made and treatment with intravenous quinidine was started. Haemoglobin levels fell from 12.9 to 5.6 g/dL. The patient was successfully transfused. The CareStart™ RDT was later performed with a result of G6PD deficiency. The Santamaria variant was revealed trough a multiplex PCR approach. This event, although very rare in the country, is significant, and indicates that treatment with anti-malarials such as PQ or quinidine could be lethal under severe G6PDd if there are no adequate conditions of care and clinical follow-up.

Poor adherence to the 14-day treatment with PQ scheme is common in many countries [44]. For that reason, the new national regulations of Honduras approved in 2017 leave the physician free to continue administering the regular treatment of PQ 0.25 mg/kg for 14 days or to choose a shortened treatment of 0.5 mg/kg for 7 days especially in the Mosquitia region, neighbouring Nicaragua, where a 7-day PQ treatment scheme is used. Given that the existence of some G6PD deficient individuals with class II deficiency in Honduras is compounded by the impossibility of the country to comply with the recommendation of the WHO to screen all newborns for G6PDd in populations with more than 3% of males affected [19], and that there is not enough capacity to implement a point-of-care test to assess the G6PD status at the national level, it would be convenient for physicians to be alert to recognize the signs of haemolysis, and be able to offer timely hospitalization for those patients who may develop severe haemolysis after PQ intake.

## Conclusions

In this study, the risk of haemolysis in patients infected with malaria was evaluated after receiving regular treatment with PQ in 3 endemic municipalities of Honduras. Seven individuals (12.7%) with genotype G6PDd A− were detected, of which 5 were hemizygous males. It was confirmed that the intake of PQ during 14 days of treatment against vivax malaria is safe in both patients with and without G6PDd, however a statistically significant difference was demonstrated in the decrease of mean haemoglobin levels among the deficient and normal patients in the 3rd and 7th days after PQ intake. Because the new national treatment scheme for vivax malaria allows the use of double dose in a shortened PQ treatment of 7 days, it is advisable to be alert for the possible cases of severe haemolysis that could occur especially among G6PD deficient males with a class II mutation.

## Additional file

**Additional file 1: Table S1.** Mean of haemoglobin concentration of individuals with G6PD genotype B and A+ by age and sex.

## Abbreviations

G6PD: glucose-6-phosphate dehydrogenase
G6PDd: glucose-6-phosphate dehydrogenase deficiency/deficient
PQ: primaquine
TQ: tafenoquine
WHO: World Health Organization
UNAH: National Autonomous University of Honduras
PCR: polymerase chain reaction
18S rRNA: 18S ribosomal RNA
RFLP: restriction fragment length polymorphism
AST: aspartate aminotransferase enzyme
ALT: alanine aminotransferase enzyme
RBC: red blood cells
RDT: rapid diagnostic test
