# Supplementary material for: G6PD deficiency, primaquine treatment, and risk of haemolysis in malaria-infected patients
Source: Malar J. 2018 Nov 8;17:415. doi: 10.1186/s12936-018-2564-2 (PMC6225638; doi:10.1186/s12936-018-2564-2)
Supplement: Supplementary file 1 — Additional file 1: Table S1. Mean of haemoglobin concentration of individuals with G6PD genotype B and A+ by age and sex. [file 12936_2018_2564_MOESM1_ESM.docx]

Table S1. Mean of haemoglobin concentration of individuals with G6PD genotype B and A+ by age and sex

|  |  |  | Mean of haemoglobin concentration g/dL | | |
| --- | --- | --- | --- | --- | --- |
| Age (years) | n | Sex | Day 1 | Day 3 | Day 7 |
| 10-18 | 7 | Male | 12.3 | 12.1 | 11.6 |
|  | 4 | Female | 10.3 | 10.1 | 11.6 |
| 19-30 | 11 | Male | 12.7 | 11.9 | 12.3 |
|  | 8 | Female | 11.7 | 11.0 | 10.9 |
| 31-40 | 6 | Male | 14.2 | 13.0 | 13.4 |
|  | 4 | Female | 12.3 | 12.0 | 12.2 |
| 41-50 | 3 | Male | 14.0 | 13.0 | 13.0 |
|  | 2 | Female | 10.6 | 9.6 | 10.2 |
| 51-74 | 1 | Male | 13.4 | 13.6 | 14.3 |
|  | 3 | Female | 11.4 | 11.1 | 11.5 |
